# Telomere length in leucocytes and solid tissues of young and aged rats

**DOI:** 10.18632/aging.203922

**Published:** 2022-02-27

**Authors:** M. Donatella Semeraro, Gunter Almer, Wilfried Renner, Hans-Jürgen Gruber, Markus Herrmann

**Affiliations:** 1 Clinical Institute of Medical and Chemical Laboratory Diagnostics (CIMCL), Medical University of Graz, Graz 8036, Austria

**Keywords:** telomeres, telomerase, shelterin, aging, Sprague Dawley rats

## Abstract

Background: Telomeres are protective nucleoprotein structures at the end of chromosomes that shorten with age. Telomere length (TL) in peripheral blood mononuclear cells (PBMCs) has been proposed as surrogate marker for TL in the entire organism. Solid evidence that supports this concept is lacking.

Methods: Relative TL (RTL) was measured in PBMCS and multiple solid tissues from 24 young (4 months) and 24 aged (14 months) Sprague-Dawley (SD) rats. The mRNA expression of telomerase (TERT) and shelterin proteins TERF-1 and TERF-2 was also measured.

Results: Mean RTL in PBMCs and solid tissues of young rats ranged from 0.64 ± 0.26 in large intestine to 1.07 ± 0.22 in skeletal muscle. RTL in PBMCs correlated with that in kidney (r = 0.315, p = 0.008), skeletal muscle (r = 0.276, p = 0.022), liver (r = 0.269, p = 0.033), large intestine (r = −0.463, p = 7.035E-5) and aorta (r = −0.273, p = 0.028). A significant difference of RTL between young and aged animals was only observed in aorta (0.98 ± 0.15 vs. 0.76 ± 0.11, p = 1.987E-6), lung (0.76 ± 0.14 vs. 0.85 ± 0.14, p = 0.024) and visceral fat (0.83 ± 0.14 vs. 0.92 ± 0.15, p = 0.44). The expression of TERT significantly differed between the tested organs with highest levels in liver and kidney. Age-related differences in TERT expression were found in PBMCs, skeletal muscle, and visceral fat. mRNA expression of TERF-1 and TERF-2 was tissue-specific with the highest levels in liver. Age-related differences in TERF-1 and TERF-2 expression were inconsistent.

Conclusions: The present study questions the utility of RTL in PBMCs as a biomarker for the individual assessment of aging.

## INTRODUCTION

Individuals age at remarkably different rates so that the health status and functional impairment can vary widely at the same chronological age [1]. Telomere length (TL) has been proposed as a biomarker of biological age that may assist to estimate individual trajectories of aging. Telomeres are protective nucleoprotein structures at the ends of eukaryotic chromosomes that are of critical importance for the preservation of our genome [2–4]. They are composed by multiple repeats of short non-coding DNA sequences and associated proteins known as shelterins. Depending on species, the basic telomeric DNA motif is six to eight base pairs long and can be found in up to several thousand copies per telomere [5]. Telomeric DNA is double-stranded for most of its length with a short single-stranded overhang at the 3′-OH end. Telomeric DNA and shelterin proteins form a unique three-dimensional structure, which is essential for the protective function of telomeres. With the help of shelterins, telomeric DNA folds backward forming a loop structure (t-loop) that allows the single stranded DNA-overhang to invade double-stranded telomeric DNA. Together, this prevents DNA repair systems from mistaking telomeric ends as DNA strand brakes and from inappropriate attempts to repair them. The complex three-dimensional structure of telomeres does also suppress the expression of genes in subtelomeric regions. This phenomenon is known as telomere positioning effect (TPE). Telomere looping does also occur over long distances so that TPEs can influence the expression of distant genes (TP-OLD) [6, 7]. Short telomeres are no longer able to maintain their complex three-dimensional structure, which abolishes TPE and TPE-OLD. As a result, formerly silenced genes become exposed and can be translated.

The progressive impairment of genomic integrity and genomic instability are key drivers of the aging process [8, 9]. With every cell division, telomeres shorten a little bit due to incomplete replication of the DNA lagging strand. In addition, accidental damage can also cause telomere shortening. This age-related shortening progressively compromises the three-dimensional structure of telomeres and once a critical threshold is reached, cells can no longer divide [10, 11]. Because of their progressive shortening, telomeres are considered a molecular clock of aging.

However, telomere shortening is not a linear process. Human and animal studies have repeatedly shown that lifestyle factors, such as obesity, physical activity, smoking, psychological stress, and sleep, modify the rate of telomere shortening. Furthermore, these factors are related to the risk of many age-related diseases and mortality. Telomerase (TERT), an enzyme that elongates telomeres, and shelterin proteins are key regulators of the telomere shortening rate. Previous studies suggest that many lifestyle factors modify the telomere shortening rate through an altered expression of these proteins.

The unique role of telomeres in the aging process has led to the idea that TL could be a useful surrogate marker of biological age. In fact, human and animal studies have shown that in age-related diseases, such as atherosclerosis, diabetes and rheumatic diseases, TL is reduced in the affected tissues [8, 12–15]. As the analysis of TL in solid tissues requires an invasive biopsy, TL in peripheral blood leucocytes (LTL) has been proposed as surrogate marker for TL in other tissues. However, solid evidence that supports this concept is largely lacking. A rather small study by Dlouha et al. analysed TL in twelve human tissues from deceased donors [16]. TL differed by factor six between different organs and LTL was only correlated to TL in liver and muscles. Besides a rather small number of cases, this study is strongly limited by a very wide age range of the donors (29 weeks to 88 years). Also, Hiam et al. reported a weak correlation between LTL and skeletal muscle TL in healthy men. However, skeletal muscle TL was not associated with age [17]. To date, the strongest evidence that supports LTL as a suitable surrogate marker for TL in other tissues is provided by the large scale GTEx project [18]. Although this study is of cross-sectional nature, the results suggest that LTL shortens at a comparable rate as other somatic cells [18]. However, specimen collection was not standardized in this study, individuals were very rather heterogeneous and information on their health status has not been obtained. A systematic mapping of TL in multiple organs and tissues of the same individual has not been performed as yet. Moreover, a structured analysis of age-related changes of TL in multiple tissues is also lacking.

Animal studies are useful to investigate age-related changes of telomeres in multiple organs of the same organism [19]. The few existing studies have focused on specific tissue types, such as leucocytes, myocardium, liver, and aorta. Similar to humans, the telomeres of murine blood leukocytes and other cell types (i.e., myocardium, liver, aorta) shorten with age [20–22], but this process may take more than a year [20, 21]. Moreover, in skeletal muscle and cardiomyocytes the age-related shortening of telomeres is accompanied by a decreased gene expression of the shelterin proteins TERF-1 and TERF-2 [22].

To address the existing gap of knowledge, the present study aimed to answer the following question. Does TL differ between leucocytes and solid organs? Is LTL correlated with TL in other organs and tissues? Does ageing induce a systematic shortening of TL in PBMCs and solid tissues? And finally, are age-related changes in TL associated with systematic changes in the expression of telomere related genes that are critical for the preservation of telomere length and structure, and telomeric function? Answering these questions will help to decide whether or not LTL is a suitable marker of biological age that represents TL in other organs of the same organism. For this purpose, we performed a systematic analysis of TL in PBMCs and multiple solid tissues from young and aged Sprague-Dawley (SD) rats. In addition, we studied the mRNA expression of telomerase (TERT) and the shelterins TERF-1 and TERF-2. All of them are established regulators of telomeric length, structure and function.

## RESULTS

### Distribution of RTL in blood leucocytes and solid organ tissues

**Figure 1:**
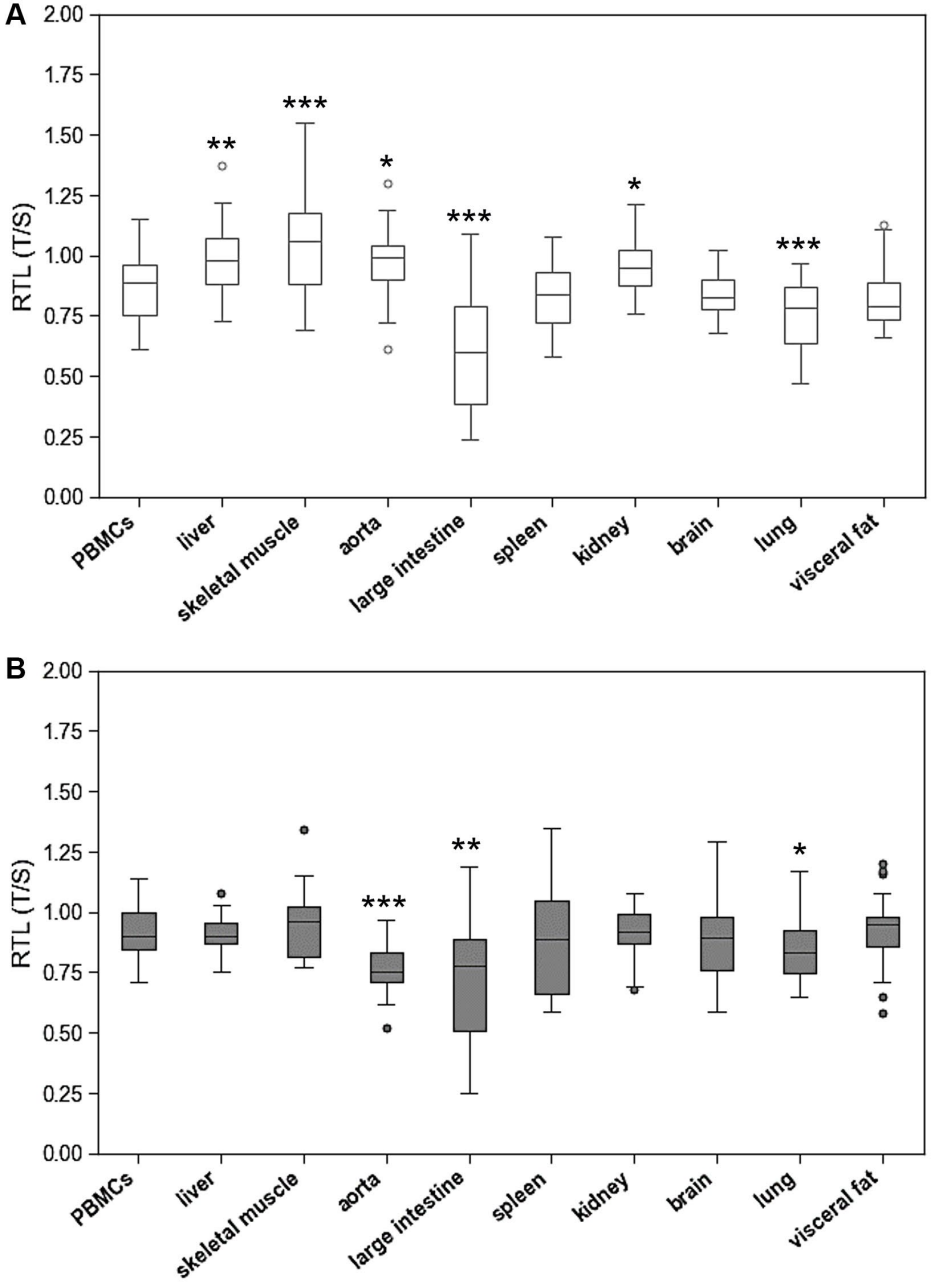
**Relative telomere length (RTL) of peripheral blood mononuclear cells (PBMCs) compared to RTL of 9 different solid organs (including liver, skeletal muscle, aorta, large intestine, spleen, kidney, brain, lung, visceral fat) isolated from.** (**A**) young and (**B**) adult rats. RTL is expressed as ratio of average telomere length to the reference gene GAPDH. ^*^p < 0.05; ^**^p < 0.01; ^***^p < 0.001 vs. PBMCs RTL.

Two out of twenty-four animals in the aged group had to be excluded from analysis due to illness. The following results describe changes in TL and expression of telomere-associated genes in the remaining 46 healthy animals. RTL showed pronounced interindividual variability across all tissues with greatest scatter in large intestine, spleen, and brain (Figure 1). The young animals had a mean RTL in PBMCs of 0.88 ± 0.15. Mean RTLs of the solid tissues ranged from 0.64 ± 0.26 in large intestine to 1.07 ± 0.22 in skeletal muscle. Liver, skeletal muscle, aorta and kidney had significantly higher mean RTLs than PBMCs. In contrast, RTL in large intestine and lung was lower than in PBMCs. Aged animals exhibited a comparable distribution of RTL across organs. Similar to young animals, mean RTL in aorta and large intestine was significantly lower than in PBMC, whereas in lung RTL was significantly higher (Figure 1).

**Figure 2:**
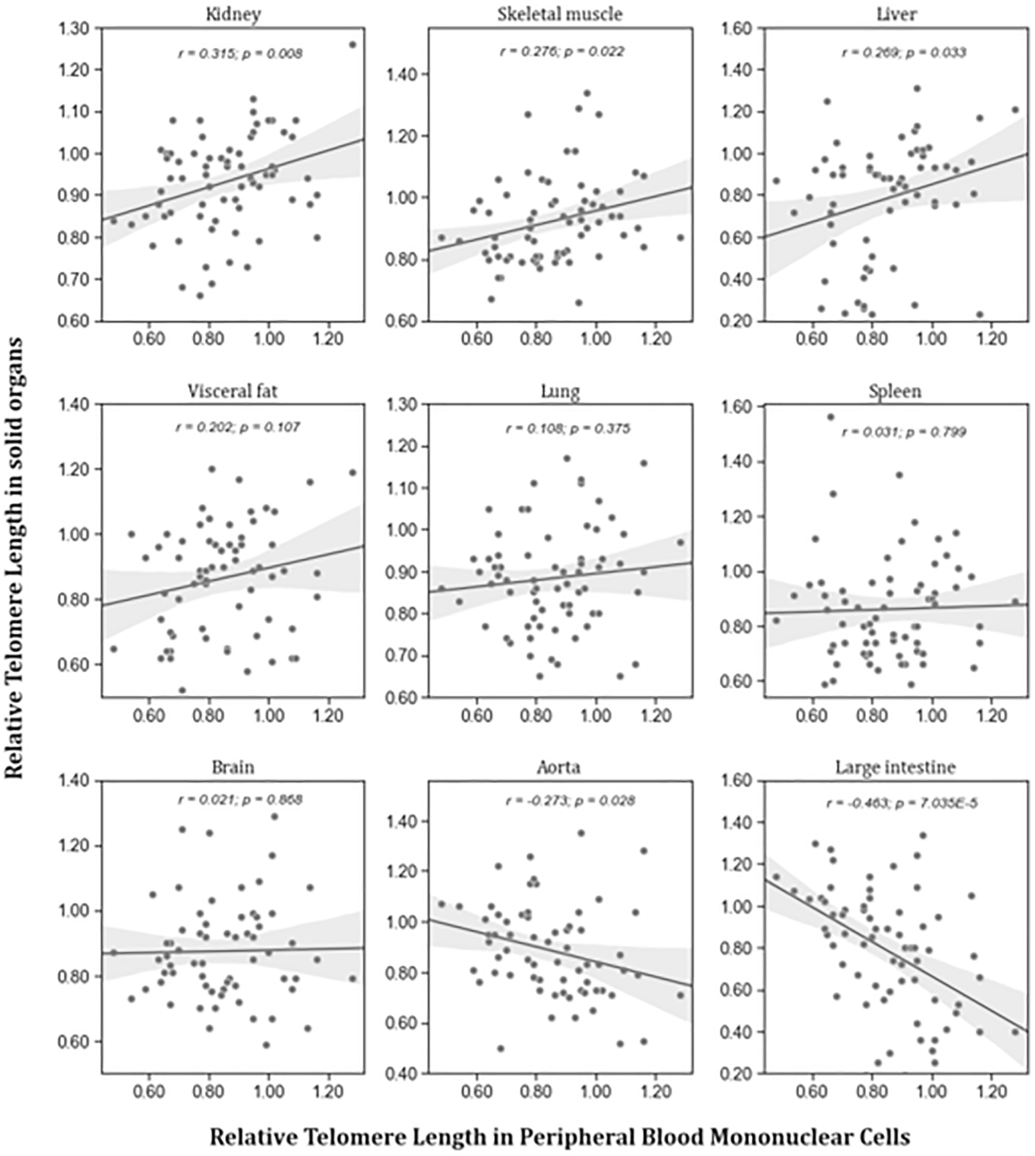
**Correlation between RTL in PBMCs and RTL in different organs isolated from adult rats (*n* = 72).** r – Pearson correlation coefficient, *p* – *p* value. The figure also shows in grey the regression line and 95% confidence interval.

To test the hypothesis that RTL in PBMCs represents the situation in other organs, RTL from PBMCs and solid organs were correlated in young and aged animals. Figure 2 shows that there was no consistent correlation between RTL in PBMCs and solid organs. Significant positive correlations were found between RTL in PBMCs and liver, skeletal muscle, and kidney. Inverse correlations were found between RTL in PBMCs and RTL in large intestine and aorta. RTL of all other tissues was not correlated to that of PBMCs.

### Age-related changes of RTL

**Figure 3:**
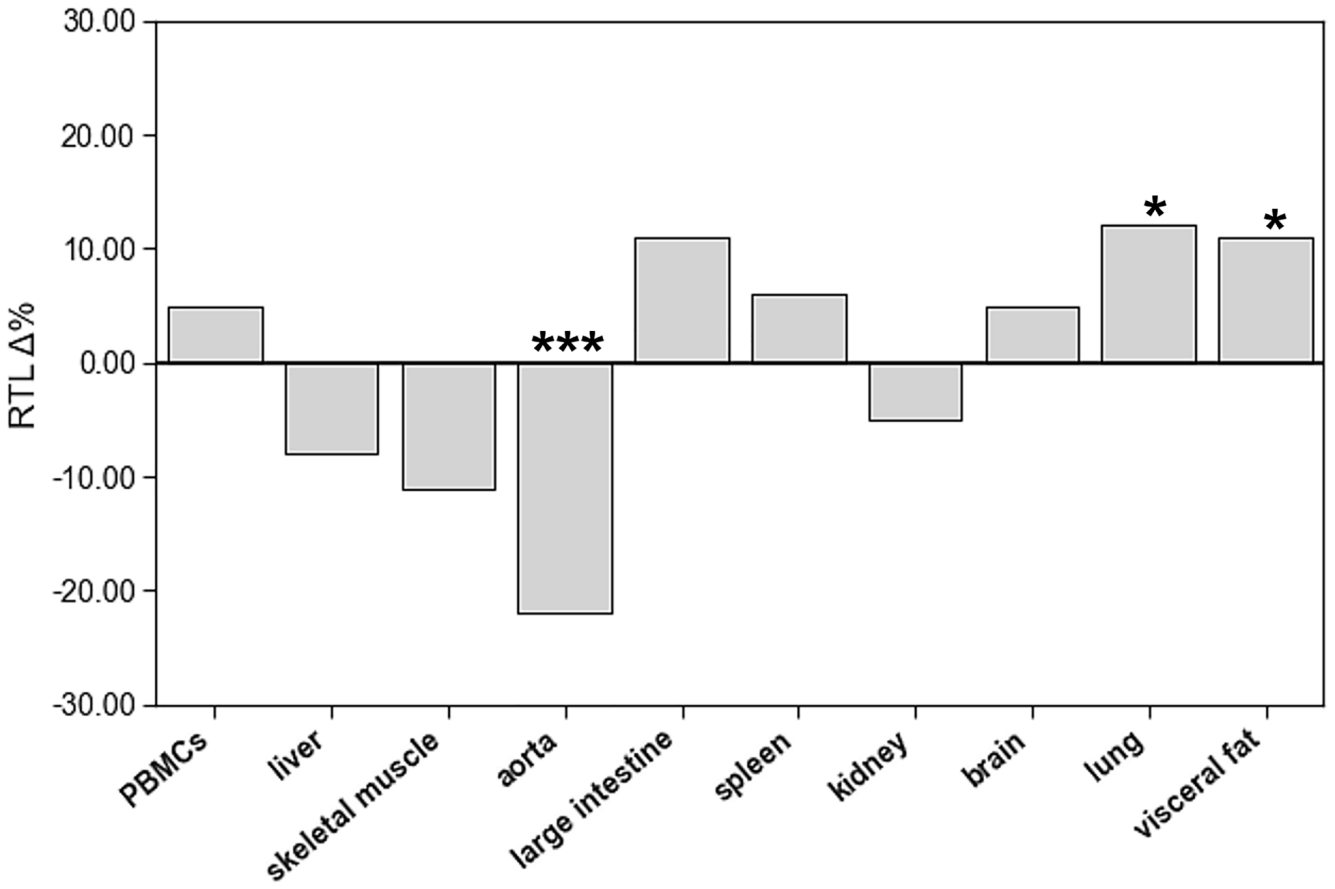
**The impact of age on relative telomere length (RTL) of different tissue types.** The boxes represent the delta% variation of RTL in adults compared to young. ^*^p < 0.05; ^***^p < 0.001 vs. young.

To identify age-related differences of RTL in PBMCs and solid tissues, we compared RTL of aged and young animals in all these matrices (Figure 3, Supplementary Table 1). The only organ with lower RTL in aged animals was aorta. Lung and visceral fat tissue showed higher RTL in aged animals. All other organs had comparable RTL in both age groups.

### Telomerase and shelterin mRNA expression from different tissue types in young and adult SD rats

**Figure 4:**
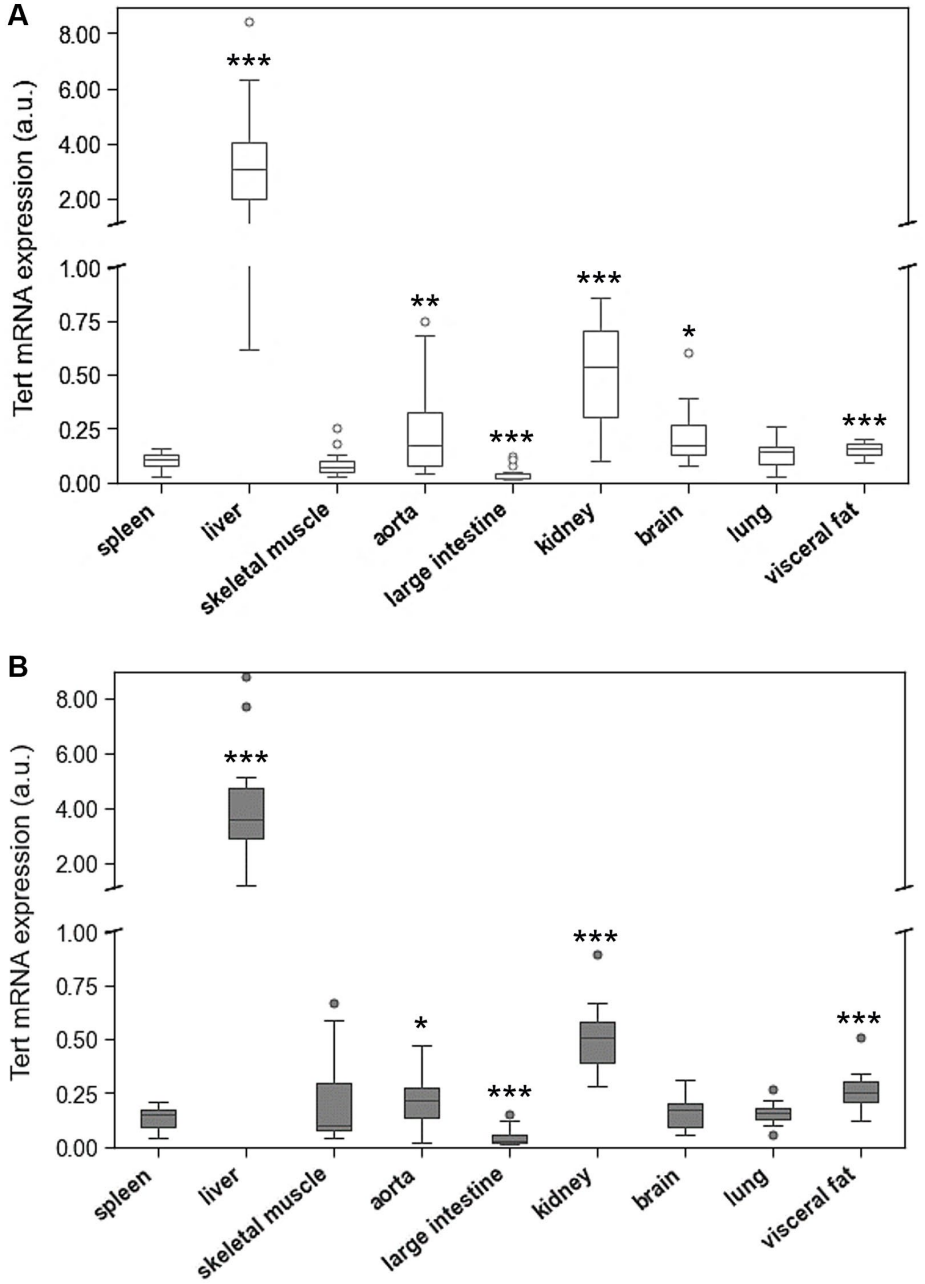
**Tert mRNA expression of spleen compared to Tert mRNA expression of 8 different solid organs (including liver, skeletal muscle, aorta, large intestine, kidney, brain, lung, visceral fat) isolated from.** (**A**) young and (**B**) adult rats. Tert mRNA expression is shown in arbitrary units. ^*^p < 0.05; ^**^p < 0.01; ^***^p < 0.001 vs. spleen Tert mRNA expression.

The expression of TERT markedly differed between the tested organs with highest levels in liver and kidney (Figure 4). In the liver, TERT mRNA expression was 40 times higher than in spleen and lung tissue.

**Figure 5:**
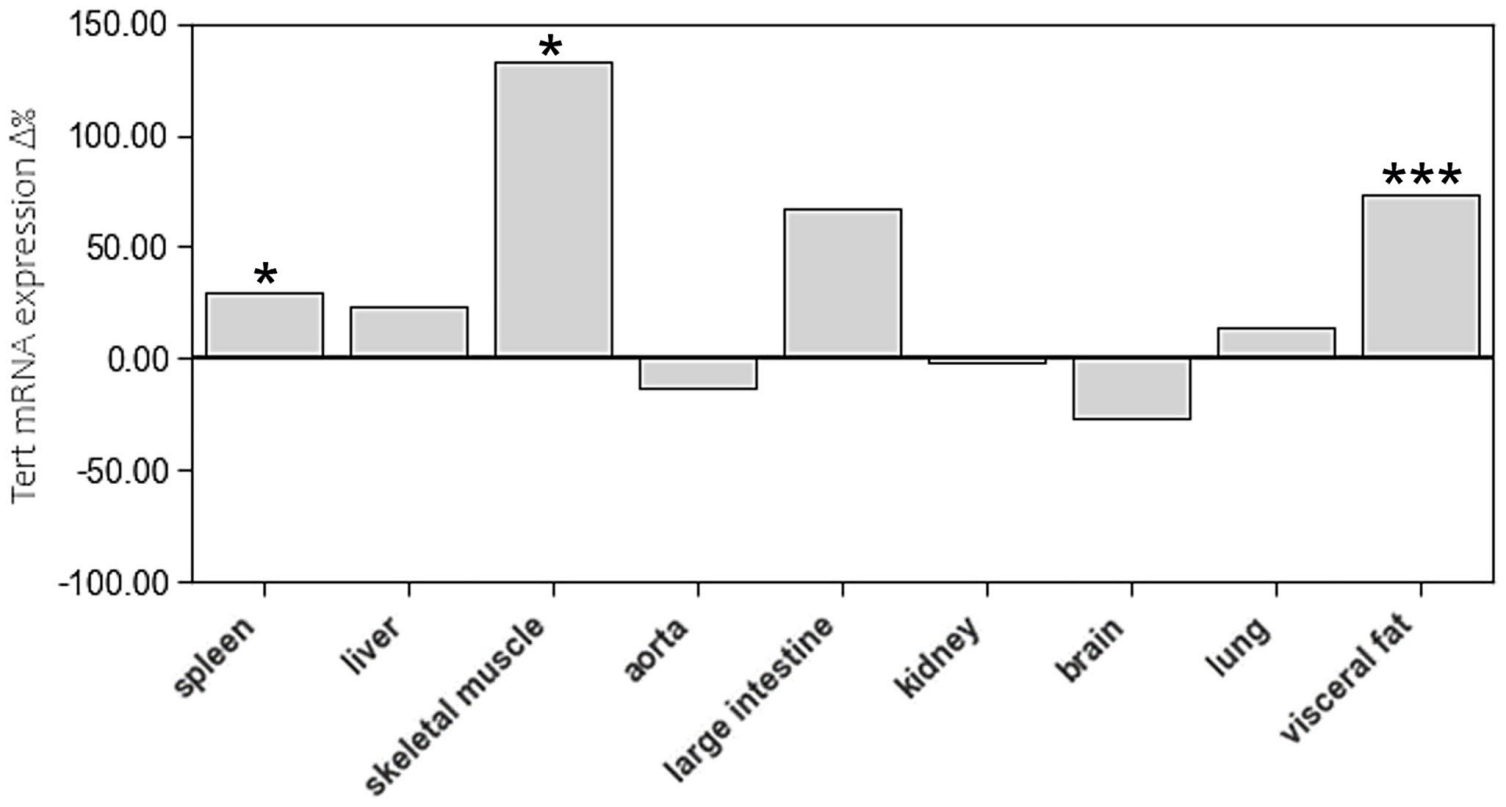
**The impact of age on Tert mRNA expression of different tissue types.** The boxes represent the delta% variation of Tert mRNA expression in adults compared to young. ^*^p < 0.05; ^***^p < 0.001 vs. young.

Age-related differences of TERT expression were only found in spleen, skeletal muscle, and visceral fat (Figure 5, Supplementary Table 1).

**Figure 6:**
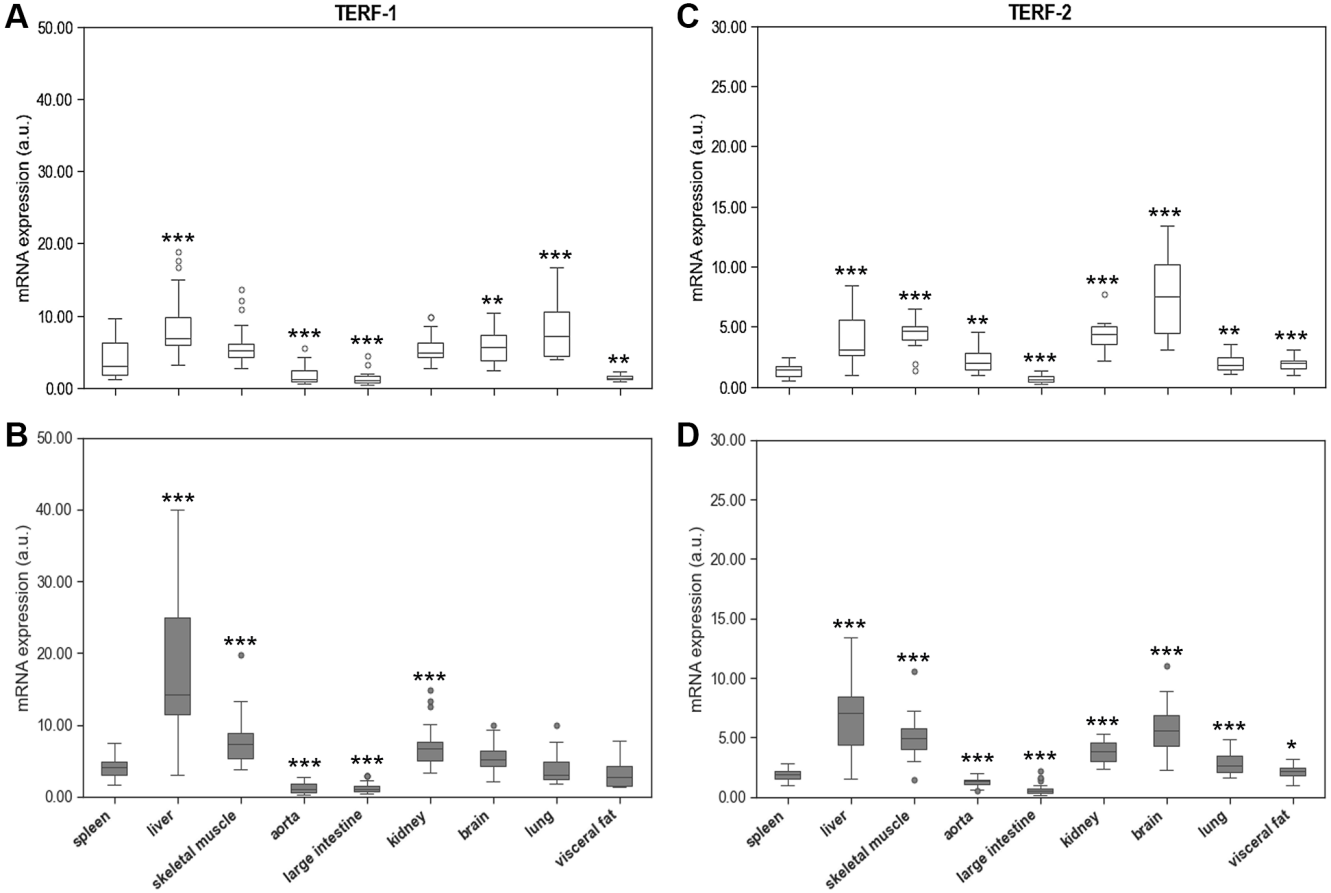
**Spleen Terf-1 mRNA expression compared to Terf-1 mRNA expression of 8 different solid organs (including liver, skeletal muscle, aorta, large intestine, kidney, brain, lung, visceral fat) isolated from.** (**A**) young and (**B**) adult rats. Spleen Terf-2 mRNA expression compared to Terf-2 mRNA expression of 8 different solid organs (including liver, skeletal muscle, aorta, large intestine, kidney, brain, lung, visceral fat) isolated from: (**C**) young and (**D**) adult rats. Terf-1 and Terf-2 mRNA expression is shown in arbitrary units. ^*^p < 0.05; ^**^p < 0.01; ^***^p < 0.001 vs. spleen Terf-1 or Terf-2 mRNA expression.

The mRNA expression of TERF-1 and TERF-2 was tissue specific showing pronounced variation between the different organs and spleen. The highest expression levels of both genes were found in liver (Figure 6). In addition, TERF-2 was highly expressed in brain. In six out of nine tissue types TERF-2 and TERT were positively associated with r = 0.801; *p* = 4,615e-11 (spleen), r = 0.560; *p* = 0.00017 (liver), r = 0.707; *p* = 1,002e-6 (aorta), r = 0.783; *p* = 1,278e-10 (large intestine), r = 0.748; *p* = 3,602e-9 (kidney), r = 0.562; *p* = 0.000106 (lung), but not in skeletal muscle, brain, and visceral fat.

**Figure 7:**
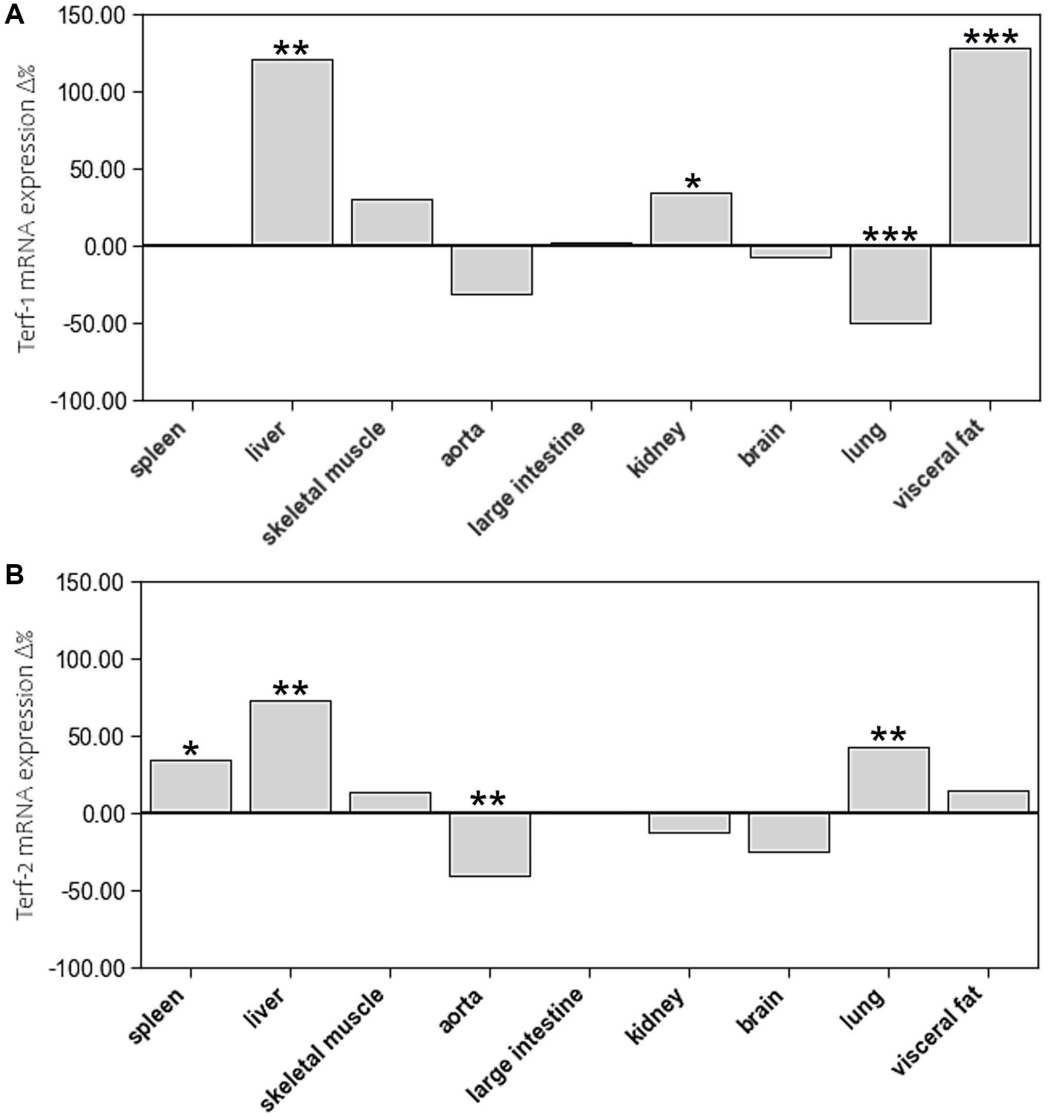
The impact of age on mRNA expression of (**A**) Terf-1 and (**B**) Terf-2 in different tissue types. The boxes represent the delta% variation of mRNA expression of Terf-1 and Terf-2 in adults compared to young. ^*^p < 0.05; ^**^p < 0.01; ^***^p < 0.001 vs. young.

Age-related differences in mRNA expression of these two shelterins were inconsistent. In aged animals, TERF-1 showed higher mRNA expression levels in liver, kidney, and visceral fat but lower levels in lung. TERF-2 expression was higher in spleen, liver, and lung of aged animals, whereas aorta showed a lower expression level (Figure 7, Supplementary Table 1).

## DISCUSSION

The systematic mapping of RTL in young and old rats showed substantial differences between organs. These differences were accompanied by pronounced interindividual variability of RTL in all tissues. Surprisingly, the distribution of RTL across different organs was comparable in young and aged animals without a systemic age-related reduction. Furthermore, correlation analyses showed no consistent association between RTL in PBMCs and solid tissues. Together, these results question the utility of RTL of PBMCs as a valid biomarker that represents the aging process in the entire organism.

Does TL between leucocytes and solid organs? The present study provides robust evidence for tissue specific RTLs. In SD rats, the longest and the shortest telomeres were found in skeletal muscle and large intestine, respectively, with a 2-fold difference. To date, similar studies have not been performed in rodents. Human studies that compared RTL between organs also found significant differences of up to 2.5-fold [16, 18]. In a rather small study by Dlouha et al, leucocytes showed the longest telomeres, whereas liver, brain and skin exhibited the shortest telomeres [16]. However, this study analysed only samples from twelve individuals with a very wide age-range of 29 weeks to 88 years, which limits the robustness of the results. In a much larger study of 6391 tissue samples from 952 donors, Demanelis et al. found the shortest telomeres in leucocytes and the longest ones in testis, skeletal muscle, and colon [18]. The organ distribution of RTL in rats and humans seems to differ substantially, which is probably due to species-effects. Despite, rigorous standardization of age, genetic background and housing conditions, the animals of the present study displayed great interindividual variation in RTL, which was similar to that observed in the human studies discussed before.

Is LTL correlated with TL in other organs and tissues? In contrast to current concepts, RTL in PBMCs was positively correlated to kidney, skeletal muscle, and liver but not to any of the other tissue types. In some tissues, such as large intestine and aorta, we even observed an inverse correlation. However, our results are aligned to those from others, Dlouha et al. also found a significant correlation of RTL in leucocytes with that in liver and muscle [16]. Moreover, in blood leucocytes and skeletal muscle biopsies of 93 healthy men between 18–87 years of age, Hiam et al. showed a weak, but significant, correlation of RTL [17]. In the large scale GTEx project, Demanelis et al. reported that RTLs were positively correlated amongst different tissues and that whole blood RTL was a reasonable surrogate for RTL in most tissues [18]. Inverse associations, as observed by Dlouha et al. in humans and us in rats, may thus be chance findings that are primarily due to the marked interindividual variability of RTL in most tissues. Based on previous studies and the present result it appears that RTL in peripheral blood cells is not a suitable biomarker to judge RTL in other organs of the same individual. However, in large cohorts there may be a robust association between RTL in peripheral blood cells and most other tissue types, which makes this an interesting marker for epidemiologic aging studies.

Does ageing induce a systematic shortening of TL in PBMCs and solid tissues? Another observation of the present study was that in most tissues RTL was comparable in young adults and aged animals. This contrasts the concept of telomere shortening with advancing age [23–26]. However, existing longitudinal studies measured RTL exclusively in leucocytes [23, 25–31]. In the large scale GTEx project, Demanelis et al. also found an age-related reduction of RTL in most tissues [18]. Here we performed a systematic analysis of RTL in multiple organs of young and aged animals that were kept under standardized conditions. Only in aortic tissue RTL was significantly lower in aged than juvenile young animals. In lung and visceral fat RTL increased with advancing age. The absence of telomere shortening in our rats could be related to their age. SD rats have a life-expectancy of 2.5 to 3.5 years. In the present study, animals were sacrificed at 14 months of age, which corresponds to mid-adult age. Therefore, it cannot be excluded that a longer aging period would have yielded a different result. Previous work from Werner et al. supports a rather slow shortening of telomeres in leucocytes and cardiomyocytes of C57/Bl6 mice [20, 21]. In these animals, a significant reduction of telomere length took up to 18 months. Although it was demonstrated that telomeric sequences are highly conserved among eukaryotic vertebrates, the length of telomeres differs between species [19]. While in humans, telomeres reach up to 20 kb in length, rodent telomere length is rather heterogeneous. For example, *Mus musculus’* telomers reach up to 150 kb in size [32]. Rats instead have telomeres ranging from 20 to 100 kb in length [33]. Therefore, the present results apply to healthy SD rats only.

Large scale human studies have also provided robust evidence that longitudinal changes of RTL vary substantially between individuals [23, 24, 26]. For example, amongst 4053 adults that were analysed at baseline and again after 7–8 years, LTL decreased in 66.3%, did not change in 11.2% and increased in 22.5% [26]. Such heterogeneous results may be due to multiple individual factors that influence telomere dynamics, such as stress, nutrition, physical activity, smoking and others [13, 34–44]. In the present animal study, most of these factors were rigorously controlled. Moreover, we analysed 10 different tissues without seeing a systematic effect. Together with existing human studies, the present results indicate that neither RTL in PBMCs nor in solid tissues is a useful marker to monitor aging on an individual level. Only in cohorts of substantial size, age-related changes of RTL can be shown reliably.

It is important to consider that existing studies have measured telomere length primarily by qPCR, which provides an average RTL across all cells and chromosomes in the sample [45]. However, there is substantial evidence that only the shortest telomeres trigger DNA damage responses and induce senescence. Information on the shortest telomeres can only be obtained by much more sophisticated methods, such as Telomere Shortest Length Assay (TeSLA) or Quantitative Fluorescence *In-Situ* Hybridization (Q-FISH). The characteristics and caveats of available methods for the analysis of telomere length have been summarized by Aubert G et al. and Lai TP et al. [45, 46]. Another important aspect is that RTL may vary within the same organ depending on the site of specimen collection. In most organs the distribution of specific cell populations is not homogenous so that regional differences may impact the results.

Are age-related changes in TL associated with systematic changes in the expression of telomere related genes? An organ specific regulation of telomeric function is further supported by the mRNA expression analyses of TERT, TERF-1 and TERF-2. Liver tissue showed by far the highest TERT mRNA expression. However, this phenomenon was not accompanied by markedly longer telomeres. Considering that hepatocytes divide rapidly, it can be assumed that the high expression of TERT is needed to prevent excessive telomere shortening in these cells. Also, TERF-1 and TERF-2 are highly expressed in hepatic tissue supporting a tissue specific maintenance of telomeres in the liver. In contrast, terminally differentiated tissues, such as skeletal muscle and brain, are characterized by a low expression level of TERT and TERF-1, but a rather high expression of TERF-2. TERF-2 is pivotal for t-loop formation and aids the invasion of the single-stranded telomeric DNA overhang into the double-stranded telomeric region upstream. Loss of TERF-2 has been shown to prevent t-loop formation and leads to excessive telomere shortening with premature cell death [47]. It can be speculated that the high TERF-2 expression in terminally differentiated tissues reflects the particular need to protect their telomeres from DDR.

In the present study, we have not seen systematic age-related differences in mRNA expression of these proteins. TERT expression was slightly higher in spleen, skeletal muscle, and visceral fat of the aged animals, but not in any of the other tissues. When considering the interindividual variability of TERT expression in most tissues, the physiological relevance of these differences is questionable. However, TERF-1 and TERF-2 expression was markedly higher in aged liver tissue, which further supports the concept of an organ specific maintenance of telomere homeostasis in rapidly dividing hepatocytes. In addition, TERF-2 expression was higher in spleen and lung tissue of aged animals, which also have a great capacity of renewal. Interestingly, aortic tissue showed a substantially lower TERF-2 expression in aged animals, which was paralleled by a reduction in RTL. Considering the rather inconclusive results of TERF-1 and TERF-2 expression, caution is warranted when interpreting them in relation to TERT and RTL. All of the above-mentioned concepts are speculative and require further research to prove them.

There are several limitations that have to be considered when interpreting the present results. Our results and conclusion exclusively apply to healthy SD rats and cannot simply be translated to the situation in various diseases or other species. A study duration of ten months with rodents is rather long but may be insufficient to capture significant age-related effects on telomeres. Considering the life expectancy of SD rats, an observation period of two years might better reflect changes in telomere biology. However, towards the end of our study, some animals started developing tumours and thus had to be excluded from the analysis. This implies that for a longer study more animals would be needed in order to obtain a sufficient number of aged animals that are free from tumours and other relevant diseases. Furthermore, we performed only one measurement per animal and tissue type, which leaves room for random effects due to regional differences in RTL. In addition, solid organ tissues cannot be collected longitudinally. Therefore, we chose a cross-sectional study design. Also, the PCR based method for RTL analysis harbours several limitations. As a relative method that requires the simultaneous measurement of a single copy reference gene, this approach is subject to greater technical variability than direct methods. Furthermore, it only provides an average TL across all telomeres. Information on the distribution of short and long telomeres can only be obtained from more sophisticated methods that are not feasible for the analysis of large sample sets. The importance of this aspect is nicely shown by a recent study from Cherif et al. that investigated gender-related lifespan differences in Wistar rats. When using a modified terminal restriction fragments (TRF) method that analysed the percentage distribution of TRFs in different ranges of restriction fragment length, they found slightly longer leucocyte telomeres in females than in males. In addition, they observed subtle gender differences in several solid organs including kidney, liver, lung, and pancreas. These differences could not be detected with the mean TRF of the traditional method [19]. Through a robust number of animals per group we aimed to compensate for most of the confounding factors. Despite rigorous standardization of the experimental conditions, this number may still be too low to demonstrate similar effects as in epidemiologic cohort studies.

## MATERIALS AND METHODS

### Animal model

Forty-eight female Sprague Dawley (SD) rats were purchased from Janvier Labs (Le Genest-Saint-Isle, France) at four months of age. All animals were fed a standard chow-based diet and kept on a 12 h/12 h light/dark cycle at the core facility animal housing at the Medical University of Graz (Austria). Temperature was maintained between 22 and 25°C. Humidity ranged between 55 to 58%. After one week of acclimatization, half of the animals were sacrificed at young age (*n* = 24). The other half was euthanatized after ten months. At the time of scarification, blood was collected by heart puncture into plasma-EDTA and serum tubes (Sarstedt, Nümbrecht, Germany) under deep isoflurane anaesthesia (Forane, Abbott, Austria). After centrifugation at 2000 g for 12 min at room temperature, plasma and serum samples were aliquoted and stored at −80°C until batched analysis. Immediately after blood collection, the following organs were explanted and snap frozen in liquid nitrogen: liver, skeletal muscle, heart, aorta, large intestine, spleen, kidney, brain, lung, visceral fat. Subsequently, all tissue samples were stored together deep-frozen at −80°C until analysis. Exclusion criteria were the development of illnesses or tumours during the intervention period. Two animals of the aged group were excluded from the study.

### Analysis of relative telomere length (RTL) in PBMCs and solid organs

After diluting 100 μl of whole blood with 100 μl of dH_2_O, DNA was isolated with the MagNA Pure LC instrument (Roche, Austria) using the Total Nucleic Isolation Kit (Roche, Austria). Subsequently, relative telomere length (RTL) of peripheral blood mononuclear cells (PBMCs) was measured by quantitative real-time PCR (qPCR) using a protocol developed by Cawthon [48].

This assay quantifies the ratio of average TL (T) to glyceraldehyde-3-phosphate dehydrogenase (GAPDH) as single copy reference gene (S). The single copy gene is used as amplification control for each sample and to determine the number of genome copies per sample. All qPCR analyses were performed on Thermocycler CFX384 TouchTM (Biorad, Germany) instrument using the following primers:

- Telomere Forward: 5′-CGGTTTGTTTGGGTTTGGGTTTGGGTTTGGGTTTGGGTT-3′;
- Telomere Reverse: 3′-GGCTTGCCTTACCCTTACCCTTACCCTTACCCTTACCCT-5′;
- GAPDH Forward: 5′-CACCTAGACAAGGATGCAGAG-3′;
- GAPDH Reverse: 3′-GCATGACTGGAGGAATCACA-5′.

All primers have been purchased from Eurofins Genomics, Austria. Each run included a standard curve made by dilutions of isolated and pooled rat DNA from 21 different blood samples, to determine the quantity of the targeted templates. RTL has been calculated as the ratio of telomere quantity to single copy reference gene quantity (T/S ratio).

RTL in solid organs was analysed with the same method described before. For this purpose, approximately 10 mg of tissue were homogenized in 300 μl Magna Pure Lysis Buffer (Roche, Austria) using the MagnaLyser (Roche, Austria). Subsequently, the DNA was isolated and quantified using the same procedure as for blood leucocytes.

### The mRNA expression analyses in blood cells and solid organs

TERT, TERF-1, and TERF-2 gene expression was analysed in RNA extracts of all solid organs. As blood leucocytes were used up for the measurement of RTL, they were not available for mRNA expression analyses. Therefore, mRNA expression in spleen was used as reference because the organ belongs to the lymphatic system and is rich in leucocytes. From each organ, 10 mg of tissue were homogenised in 300 μl Magna Pure Lysis Buffer (Roche, Austria) using the MagnaLyser (Roche, Austria). RNA was extracted from these homogenates with the Total Nucleic Isolation Kit (Roche, Austria) on a MagNA Pure LC instrument (Roche, Austria). Subsequently, the mRNA in these extracts was transcribed into cDNA using the QuantiTect Reverse Transcription kit (Qiagen, Germany). Finally, mRNA expression of TERT, TERF-1, and TERF-2 was analysed by qPCR with TaqMan probes (Life Technologies dba Invitrogen, United States). The expression of each target gene expression was calculated with the ΔΔCT method using β-actin as reference gene. The sequences of the probes used were as follows:

- Β-actin: 5′-CTTCCTTCCTGGGTATGGAATCCTG-3′;
- Tert: 5′-ATCGAGCAGAGCATCTCCATGAATG-3′;
- Terf-1: 5′-AAAACAGACATGGCTTTGGGAAGAA-3′;
- Terf-2: 5′-GAGAAAATTTAGACTGTTCCTTTGA-3′.

### Statistical analyses

Results are shown as mean ± standard deviations. Group differences were assessed using the two-tailed Student’s *t* test for dependent or independent samples or the Mann-Whitney *U* test depending on the distribution of the data. Correlations between variables were determined by linear regression analysis according to Pearson (r, Pearson Correlation coefficient; p, univariate ANOVA). *p* values of <0.05 were considered statistically significant. Data were plotted using Python programming language with Jupyter Notebook within the data science package Anaconda3 for Windows. IBM SPSS v. 26 for Windows was used for explorative data analysis and a level of acceptance of the null hypothesis was set at *p* = 0.05.

### Institutional review board statement

The study was approved by the responsible National Ethics Committee (GZ: 66.010/0070-V/3b/2018) and conducted in accordance with the guidelines of the Animal Care and Use Committee of the Ministry of Science and Research, Vienna, Austria.

### Data availability statement

The datasets generated during and/or analysed during the current study are based on the work for a PhD thesis and therefore are not publicly available but are available from the corresponding author on reasonable request.

## CONCLUSIONS

In conclusion, the present study questions the utility of RTL in PBMCs as a biomarker for the individual assessment of aging. Despite rigorous standardization of housing conditions, sample collection and analytical procedures, excessive intra- and interindividual variability has been observed. Furthermore, RTL in PBMCs was not systematically correlated with solid organ tissues and no differences have been observed between young and old animals. Thus, future studies should focus on the analysis of older animals and measurement of the shortest telomeres.

## Supplementary Materials

Supplementary Table 1
