# Supplementary material for: Telomere length in leucocytes and solid tissues of young and aged rats
Source: Aging (Albany NY). 2022 Feb 27;14(4):1713–28. doi: 10.18632/aging.203922 (PMC8908913; doi:10.18632/aging.203922)
Supplement: Supplementary Table 1 [file aging-14-203922-s001.pdf]

SUPPLEMENTARY TABLE

Supplementary Table 1. Relative telomere length, telomerase and shelterin gene expression in PBMCs and solid organs.

	RTL		*TERT* *mRNA expression*		*TERF-1* *mRNA expression*		*TERF-2* *mRNA expression*	
Cell/Tissue	**Young**	**Old**	**Young**	**Old**	**Young**	**Old**	**Young**	**Old**
PBMCs	0.88 ± 0.15	0.92 ± 0.11						
Spleen	0.83 ± 0.14	0.88 ± 0.21	0.11 ± 0.03	0.13 ± 0.06*	4.21 ± 2.91	4.26 ± 1.65	1.38 ± 0.53	1.85 ± 0.53$^{*\$\$\$}$
Liver	0.99 ± 0.16$^{\$\$}$	0.91 ± 0.09	3.55 ± 1,92$^{\$\$\$}$	3.99 ± 2.33$^{\$\$\$}$	8.52 ± 4.50$^{\$\$\$}$	19.08 ± 15.30$^{**\$\$\$}$	3.95 ± 2.24$^{\$\$\$}$	6.84 ± 3.24$^{**\$\$\$}$
Kidney	0.96 ± 0.13$^{\$}$	0.91 ± 0.11	0.52 ± 0.23$^{\$\$\$}$	0.5 ± 0.12$^{\$\$\$}$	5.46 ± 2.01	7.54 ± 3.13$^{*\$\$\$}$	4.29 ± 1.19$^{\$\$\$}$	3.76 ± 1.00$^{\$\$\$}$
Muscle	1.08 ± 0.30$^{\$\$\$}$	0.96 ± 0.14	0.09 ± 0.05	0.21 ± 0.20*	6.12 ± 2.88	7.89 ± 3.63$^{\$\$\$}$	4.46 ± 1.18$^{\$\$\$}$	5.04 ± 1.82$^{\$\$\$}$
Aorta	0.98 ± 0.15$^{\$}$	0.76 ± 0.11$^{***\$\$\$}$	0.24 ± 0.20$^{\$\$}$	0.2 ± 0.12$^{\$}$	1.83 ± 1.30$^{\$\$\$}$	1.28 ± 0.75$^{\$\$\$}$	2.23 ± 1.06$^{\$\$}$	1.35 ± 0.37$^{**\$\$\$}$
Intestine	0.64 ± 0.26$^{\$\$\$}$	0.71 ± 0.27$^{\$\$}$	0.04 ± 0.03$^{\$\$\$}$	0.05 ± 0.04$^{\$\$\$}$	1.24 ± 0.91$^{\$\$\$}$	1.25 ± 0.77$^{\$\$\$}$	0.68 ± 0.28$^{\$\$\$}$	0.70 ± 0.54$^{\$\$\$}$
Brain	0.84 ± 0.09	0.88 ± 0.17	0.21 ± 0.16$^{\$}$	0.16 ± 0.08	5.93 ± 2.19$^{\$\$}$	5.28 ± 2.00	7.59 ± 3.34$^{\$\$\$}$	5.79 ± 2.18$^{\$\$\$}$
Lung	0.76 ± 0.14$^{\$\$\$}$	0.85 ± 0.14$^{*\$}$	0.14 ± 0.07	0.16 ± 0.04	8.33 ± 4.20$^{\$\$\$}$	4.19 ± 2.30***	1.99 ± 0.69$^{\$\$}$	2.79 ± 1.01$^{**\$\$\$}$
Visceral fat	0.83 ± 0.14	0.92 ± 0.16*	0.15 ± 0.03$^{\$\$\$}$	0.26 ± 0.09$^{***\$\$\$}$	1.4 ± 0.41$^{\$\$}$	3.26 ± 2.03***	1.93 ± 0.46$^{\$\$\$}$	2.22 ± 0.56$^{\$}$

Data are presented as means ± SD; significant differences were highlighted between young and adult animals (*), and vs. PBMCs for RTL ($^{\$}$) or spleen for gene expression analysis ($^{\$}$) in either young or adult animals. $^{*}p < 0.05$, $^{**}p < 0.01$, $^{***}p < 0.001$ vs. young; $^{\$}p < 0.05$, $^{\$\$}p < 0.01$, $^{\$\$\$}p < 0.001$ vs. reference tissue.
